# Agonistic Human Antibodies Binding to Lecithin-Cholesterol Acyltransferase Modulate High Density Lipoprotein Metabolism*

**DOI:** 10.1074/jbc.M115.672790

**Published:** 2015-12-07

**Authors:** Ruwanthi N. Gunawardane, Preston Fordstrom, Derek E. Piper, Stephanie Masterman, Sophia Siu, Dongming Liu, Mike Brown, Mei Lu, Jie Tang, Richard Zhang, Janet Cheng, Andrew Gates, David Meininger, Joyce Chan, Tim Carlson, Nigel Walker, Margrit Schwarz, John Delaney, Mingyue Zhou

**Affiliations:** From ‡ Therapeutic Discovery, Amgen Inc., Seattle, Washington 98119,; § Cardiometabolic Disorders,; ¶ Therapeutic Discovery, and; ** PKDM Department, Amgen Inc., South San Francisco, California 94080, and; ‖ Therapeutic Discovery, Amgen Inc., Burnaby, British Columbia V5A 1V7, Canada

**Keywords:** antibody engineering, cholesterol metabolism, drug discovery, enzyme, high density lipoprotein (HDL)

## Abstract

Drug discovery opportunities where loss-of-function alleles of a target gene link to a disease-relevant phenotype often require an agonism approach to up-regulate or re-establish the activity of the target gene. Antibody therapy is increasingly recognized as a favored drug modality due to multiple desirable pharmacological properties. However, agonistic antibodies that enhance the activities of the target enzymes are rarely developed because the discovery of agonistic antibodies remains elusive. Here we report an innovative scheme of discovery and characterization of human antibodies capable of binding to and agonizing a circulating enzyme lecithin cholesterol acyltransferase (LCAT). Utilizing a modified human LCAT protein with enhanced enzymatic activity as an immunogen, we generated fully human monoclonal antibodies using the XenoMouse^TM^ platform. One of the resultant agonistic antibodies, 27C3, binds to and substantially enhances the activity of LCAT from humans and cynomolgus macaques. X-ray crystallographic analysis of the 2.45 Å LCAT-27C3 complex shows that 27C3 binding does not induce notable structural changes in LCAT. A single administration of 27C3 to cynomolgus monkeys led to a rapid increase of plasma LCAT enzymatic activity and a 35% increase of the high density lipoprotein cholesterol that was observed up to 32 days after 27C3 administration. Thus, this novel scheme of immunization in conjunction with high throughput screening may represent an effective strategy for discovering agonistic antibodies against other enzyme targets. 27C3 and other agonistic human anti-human LCAT monoclonal antibodies described herein hold potential for therapeutic development for the treatment of dyslipidemia and cardiovascular disease.

## Introduction

Atherosclerosis leads to the clinical manifestation of cardiovascular disease (CVD),^2^ the number one cause of death in the developed world. Mortality caused by atherosclerotic coronary artery disease is expected to remain high even with statins and ezetimibe being used as a standard of care and a new antibody therapy against the proprotein convertase subtilisin/kexin 9 reaching the market (1). A wealth of observational data accrued in a variety of clinical settings over several decades suggests that modulating high density lipoprotein (HDL) metabolism may be a viable therapeutic strategy for complementing low density lipoprotein (LDL)-lowering treatments (2). The sizable unmet medical need has driven intensive drug discovery and development activities to target an array of factors that regulate HDL metabolism, including apolipoprotein (apoA-I) and cholesteryl ester transfer protein (CETP) (3). However, clinical trials and failures over the past several years in these arenas suggest that HDL therapeutic approaches need to go beyond simply raising circulating HDL cholesterol (HDL-C) levels. Importantly, modulating HDL metabolism by well defined mechanisms of action to promote efflux of cholesterol from existing atherosclerotic plaque lesions in the vessel walls is a key consideration for target validation, biomarker evaluation, and proof of concept (4).

LCAT (EC 2.3.1.43) is one of the key factors that impacts HDL metabolism. It is the only enzyme in the blood that catalyzes esterification of free cholesterol (FC) to form cholesteryl ester (CE) and lipidates apoA-I and HDL (5). By converting FC into CE, which subsequently is sequestered to the core of HDL particles for further transport and metabolism, LCAT plays an essential role in the formation and maturation of HDL particles as well as in the maintenance of plasma levels of apoA-I and HDL-C (6). Not only does LCAT promote generation of larger and spherical α-HDL particles; its enzymatic activity creates an irreversible gradient of FC between peripheral tissues and HDL particles in both the blood and tissue liquids. As a result, LCAT facilitates the transfer of cholesterol from peripheral tissues and cell membranes to apoA-I and HDL particles (7). With this role, the LCAT activity provides a driving force for reverse cholesterol transport (RCT), a pathway that describes flux of cholesterol from peripheral tissues to the liver for excretion (8). By promptly and appropriately lipidating apoA-I, LCAT activity also prevents loss of the lipid-free apoA-I and small HDL particles via kidney filtration. These activities distinguish LCAT from several other HDL-regulating factors, including CETP.

Evidence that supports LCAT activity in driving RCT and preventing atherogenesis has evolved from a number of preclinical studies in which LCAT activity was increased by various means in animals expressing *CETP* (hamsters, rabbits, or monkeys). For instance, rabbits with *LCAT* overexpression showed strong resistance to developing atherosclerosis when fed a high cholesterol diet (9). In another study, transgenic rabbits that lacked either one or both copies of a functional LDL receptor revealed that LCAT may have the ability to affect atherosclerosis through the LDL receptor pathway (10). In addition, adenovirus-mediated *LCAT* overexpression in rabbits was associated with a roughly 2-fold increase in HDL-C, inhibition of atherosclerosis, and increased cholesterol unloading from atherosclerotic lesions (11). Furthermore, adenovirus-mediated *LCAT* gene transfer to hamsters led to increased cholesterol excretion in feces (12). Studies performed in rodent species that lack CETP showed inconsistent results with LCAT intervention, presumably because CETP plays a role in the RCT pathway at a step immediately downstream of LCAT action to transfer CE from HDL to the apoB-containing lipoproteins toward the liver portal (13).

Human genetic data from several independent genome-wide studies confirm an association between SNPs in the *LCAT* gene and dyslipidemia (14–16). On the other hand, studies of CVD in patients with genetic LCAT deficiency were inconclusive. LCAT genetic deficiency leads to two rare autosomal recessive disorders, familial LCAT deficiency (17) and fish eye disease (18). Both phenotypes exhibit very low plasma HDL-C levels and corneal opacities. Renal failure appears to be the major cause of morbidity and mortality in these patients. CVD risk has been reported in both types of LCAT-deficient patients (19), but in several cases, clinical CVD manifestations are not readily apparent and are perhaps complicated by reduced LDL-cholesterol levels associated with LCAT deficiency in these cases. A study of 47 heterozygotes for *LCAT* gene mutations revealed low plasma HDL-C levels, elevated triglycerides, and high sensitive C-reactive protein levels and increased carotid intima media thickness (20). Similar findings were reported from an unrelated LCAT-deficient cohort in Canada (21) and 13 Italian families (22). Interestingly, the same 13 Italian families were evaluated again 4 years later, but carotid intima media thickness measurement in 40 patients with LCAT deficiency was not increased compared with unrelated control subjects selected from a blood donor database (23). Potential limitations of this study include differences in ultrasound methodology within the carrier cohort and lack of a familial relationship of the controls with the carriers.

Several epidemiological and clinical studies in the general population revealed a correlation between plasma LCAT enzyme activity and CVD risk. A 48–58% decrease of plasma LCAT activity was associated with acute myocardial infarction and CVD in 90 patients (24). Another study in subjects with ischemic heart disease revealed a strong association of ischemic heart disease with low plasma LCAT activity, suggesting that LCAT activity might be useful as a biomarker for identifying patients at risk for CVD (25). A few other studies failed to capture an association between low LCAT levels and increased atherosclerosis or, conversely, an association of increased LCAT activity with lower CVD risk (26, 27). Limitations of these studies include a narrow window of LCAT activity variation among the cohorts and lack of a robust and consistent methodology in clinical laboratories for determining LCAT enzyme activity in serum samples.

We propose that LCAT activation represents a novel therapeutic strategy for the treatment of dyslipidemia and atherosclerosis through the mechanism of favorably modulating HDL metabolism and promoting RCT. Experimental evidence in support of this notion includes our previous study in which supplementation of a modified recombinant LCAT protein accelerated cholesterol mobilization *in vivo* and attenuated atherogenesis in rabbits fed with a high cholesterol diet (28). In addition, we found that the endogenous LCAT enzyme can be activated by a synthesized small molecule (29). We reasoned further that endogenous LCAT in the bloodstream could be activated by an agonistic antibody, which would lead to the desirable pharmacological effectiveness. This study established a novel strategy for successful discovery of agonistic antibodies for human LCAT and demonstrated the feasibility of developing a human anti-human LCAT antibody therapy with favorable pharmacodynamic and pharmacokinetic properties in non-human primates.

## Experimental Procedures

### 

#### 

##### Generation of Recombinant Human and Cynomolgous Monkey LCAT Proteins

Full-length human LCAT-coding region was cloned from liver and brain cDNAs (BioChain Institute, Inc., Hayward, CA) and subcloned into the expression vector pDSRa25. Mutagenesis was performed using a QuikChange site-directed mutagenesis kit (Stratagene) according to the manufacturer's protocol. Recombinant human wild type LCAT proteins (rLCAT) were generated from a serum-free, suspension-adapted Chinese hamster ovary (CHO) DXB-11 cell line designated CHO/CS9. Production cultures were seeded in serum-free culture medium with 0.5 mm sodium butyrate at 2.0 × 10^6^ cells/ml in 125-ml shake flasks and incubated at 31 °C for 5 days. Purification of rLCAT was performed using an anti-human LCAT affinity column generated by cross-linking in-house generated mouse anti-human LCAT 9B14.A2 monoclonal antibody to a Protein A affinity medium, MabSelect SuRE (GE HealthCare). For generation of cynomolgus monkey LCAT proteins, the full-length cynomolgus LCAT-coding region was cloned from cynomolgus liver cDNAs (BioChain Institute, Inc., Hayward, CA), and was constructed into the expression vector pTT5 with a His_6_ tag at the C terminus of the recombinant protein. Purification of cynomolgus LCAT proteins (cynoLCAT) was performed by concentrating cynoLCAT-His_6_-containing cell culture supernatant on a Millipore PrepScale TFF 10,000 molecular weight cut-off cartridge and buffer-exchanging into phosphate-buffered saline (PBS), pH 7.2. Concentrated supernatant was then loaded onto a 5-ml HisTrap FF (GE HealthCare) column equilibrated with a buffer containing 20 mm sodium phosphate, 300 mm NaCl, and 20 mm imidazole (pH 7.2). After loading, the column was washed with the equilibrating buffer, and cynoLCAT was eluted with a 5CV gradient to 300 mm imidazole in the equilibrating buffer. Subsequent gel filtration purification was performed using HiLoad Superdex 200 26/60 equilibrated in formulation buffer (PBS, 10% glycerol, 50 μm EDTA, pH 7.2) to isolate purified proteins.

##### Antibody Generation and Screening

Monoclonal antibodies were generated using the XenoMouse^TM^ technology according to the methods disclosed in U.S. Patent Application 08/759,620 (30) and International Patent Applications WO 98/24893 (31) and WO 00/76310 (32). Soluble rLCAT and a modified human LCAT protein with a single point mutation, C31Y, designated as rLCAT(C31Y), were used as immunogens. Following the initial immunization, subsequent boost immunizations of immunogen (5 μg/mouse of soluble LCAT proteins) were administered on a schedule and for the duration necessary to induce a suitable titer of anti-LCAT antibody resulting in a total of 12 immunizations over a period of approximately 2 months, which generated a panel of 23,000 hybridomas. Lymphocytes from animals exhibiting suitable titers were obtained from draining lymph nodes. B cells were selected from this population and fused with non-secretory myeloma fusion partner P3X63Ag8.653 cells (American Type Culture Collection CRL 1580) (33) using PGE/DMSO (Sigma-Aldrich). The fused cells were gently pelleted and resuspended in selection medium containing azaserine and hypoxanthine and cultured for 3 to 4 days. Cells were distributed into 96-well plates using standard techniques to maximize clonality of the resulting colonies. After several days of culture, supernatants were collected and subjected to screening assays as detailed below, including confirmation of binding to human LCAT, cross-reactivity with LCAT of other species (cynomolgus monkey, rabbit, and murine), and ability to agonize LCAT enzymes. Positive cells were further selected and subjected to standard cloning and subcloning techniques. Clonal lines were expanded for generating hybridoma culture supernatants.

The primary binding screen to identify human LCAT binders was performed by examining the hybridoma culture supernatants using ELISA as follows. Microtiter plates (Costar 3702) were incubated with neutravidin (Thermo 31000) at 10 μg/ml and with a volume of 40 μl/well overnight at 4 °C. The wells were washed for three cycles with 1× PBS and then blocked with 1% nonfat skim milk in 1× PBS (90 μl/well) for at least 30 min at room temperature. After wash, 40 μl of 1 μg/ml biotinylated rLCAT was added to each well and incubated for 1 h at room temperature. The wells were washed as before, and hybridoma culture supernatants were added to wells diluted in 1× PBS containing 1% nonfat skim milk for a final concentration of 20% and incubated for 1 h at room temperature. After washing, goat anti-human IgG Fc HRP was added at 100 ng/ml in 1× PBS containing 1% nonfat skim milk at 40 μl/well and incubated for 1 h at room temperature. The absorbance at 450 nm was read after adding 40 μl/well 1 m hydrochloric acid.

Primary activity screening for identification of human anti-human LCAT-agonistic antibodies was performed using a scintillation proximity assay (SPA) to first filter out LCAT antagonistic antibodies. This SPA used 0.5 μl of hybridoma culture supernatant samples to incubate with rLCAT(C31Y) at a concentration of 0.1 μg/ml protein in the reaction mixture containing 1% human serum albumin (fatty acid-free), 7 mm Tris (pH 7.4), 100 mm NaCl, and 2 mm β-mercaptoethanol. The LCAT reaction substrates consisted of human apoA-I proteoliposomes containing 210 μm l-α-phosphatidylcholine type XVI-E (Sigma), 13 μm cholesterol, 28 μm [1,2-^3^H]cholesterol (PerkinElmer Life Sciences), and 56 μg/ml purified human apoA-I protein (Meridian Life Sciences). Reactions were conducted in quadruplicate in 384-well plates and incubated at room temperature for 5 h. LCAT activity on cholesterol esterification was determined by adding 25 mg/ml 2-OH-β-cyclodextrin to block non-reacted cholesterol and then adding 5 mg/ml Ysi-SPA beads to bind and detect CE-contained proteoliposomes. The signal from the SPA beads was quantified using a TopCount (PerkinElmer Life Sciences) plate reader. Antibody containing hybridoma exhaust supernatants that did not bind to LCAT were used as non-inhibiting controls. Hybridoma samples with activity above one S.D. of average control activity were selected as “non-antagonistic” antibodies and subjected to confirmation assays to further identify agonistic antibodies.

##### LCAT Activation Assays

The non-antagonistic antibodies selected by the SPA assay were examined using another LCAT activity assay with thin layer chromatography (TLC) to quantify the rate of cholesterol esterification in reconstituted HDL particles (34). For this assay, 10 μl of hybridoma exhaust supernatants were incubated with purified wild type rLCAT protein at a concentration of 0.8 μg/ml protein in the presence of 1% human serum albumin, 7 mm Tris (pH 7.4), 4 mm EDTA, 100 mm NaCl, and 2 mm β-mercaptoethanol. LCAT reaction substrates consisted of human apoA-I proteoliposomes containing 67 μm l-α-phosphatidylcholine type XVI-E, 8 μm cholesterol, 4 μm [4-^14^C]cholesterol (PerkinElmer Life Sciences), and 12 μg/ml apoA-I. Reactions were run in triplicate and incubated in a 37 °C water bath for 1 h. Following the reaction, lipids were extracted with a 10-fold volume addition of 100% ethanol. Lipids containing supernatants were obtained by centrifugation and then dried under a stream of nitrogen gas, resuspended in chloroform, and spotted on TLC plates (Silica Gel 60, Whatman). FC and CE were separated by running plates in a TLC chamber with petroleum ether/ether/acetic acid (100:20:0.5, v/v/v). FC and CE bands were detected by exposing to image plates and subsequent reading on a FujiFilm FLA-5100 fluorescent image analyzer. Ratios of FC/CE were determined using ImageQuant software (Fujifilm). Agonistic activity was confirmed in selected medium samples by a volumetric dose response using 0, 0.4, 2, and 10 μl with the same assay conditions. Antibodies that showed dose-dependent LCAT-agonizing activity with an increased rate of cholesterol esterification in this assay were reconfirmed upon purification in dose-titration experiments.

##### LCAT-Phospholipase A_2_ Activity Assays

Characterization of identified agonistic antibodies upon modulating the phospholipase A_2_ activity of LCAT (LCAT-PLA_2_) was performed using a commercially available LCAT-phospholipase activity assay kit (Millipore). The kit provides non-hydrolyzed substrates that, upon hydrolysis by LCAT-PLA_2_ activity, generate fluorescence signals at 390 nm. rLCAT protein was preincubated with identified antibodies prior to the incubation with this substrate. LCAT-PLA_2_-mediated hydrolysis was assessed by measuring the emission at 390 nm after 4 h of incubation with rLCAT-antibody complex at 37 °C. Two concentrations of antibodies (1 and 10 nm) were tested with two different rLCAT concentrations (1 and 10 nm) in the reactions. A small molecule agonist of LCAT was used at 3 μm as a control (29, 35).

##### Sequence Analysis and Cloning of Agonistic Antibodies

Hybridoma sequencing was performed by preparing total RNA from the hybridoma cells by using the RNeasy 96 kit (Qiagen). An aliquot of total RNA was used in RT-PCR to amplify the heavy chain (HC) and light chain (LC) of the antibody using the Qiagen OneStep RT-PCR kit. The antibody LC was amplified using an antisense primer to anneal to the 5′ region of the human λ chain constant region and a sense primer to anneal to the 5′-UTR region of human λ light chain genes. For antibody 27C3, the HC of the antibody was amplified using an antisense primer to anneal to the 5′ region of CH1 region of IgG constant genes and a sense primer to anneal to the 5′-UTR region of VH4 V genes. For antibody 18E5, the LC was amplified using an antisense primer to anneal to the 5′ region of human κ constant region and a sense primer to anneal to the first 7–8-amino acid region of κ leader genes. The HC of 18E5 was amplified using an antisense primer to anneal to the 5′ region of CH1 region of IgG constant genes and a sense primer to anneal to the first 7–8-amino acid region of VH V gene leaders. The amplified cDNAs were gel-purified and sequenced using the antisense primer used in their respective PCR, and the sequence was confirmed using gene-specific sense primers. Amino acid sequences were then deduced from the nucleic acid sequences.

##### Generation of Recombinant Cloning of Agonistic Antibodies and Stable CHO Cell Lines

Stable CHO cell lines were created using mammalian expression vectors licensed from Selexis SA (Geneva, Switzerland). The HC and LC of 27C3 were cloned into pSUREtech153 vector, which contains an hGAPD promoter and puromycin selection marker at the SalI/NotI site. The plasmid has an hGAPD promoter and puromycin selection marker. TOP10 *Escherichia coli* cells were transformed with each vector and mixed with 100 ml of Terrific broth in a 250-ml baffled flask with overnight shaking at 220 rpm. Large scale plasmid purifications were done using the BenchPro 2100 (Life Technologies, Inc.) according to the manufacturer's protocol. Suspension-adapted CHO-K1 (ATCC® CCL-61^TM^) cells were transfected with LipofectamineLTX (Life Technologies) using a 1:1 ratio of expression vectors, normalized by mass. Transfected cells were cultured and selected using 10 μg/ml puromycin and 600 μg/ml hygromycin B. Cells were cultivated in suspension format using disposable shake flasks and placed in a humidified incubator (37 °C, 5% CO_2_), rotating at 150 rpm. The selection medium was replaced every 3–4 days for ∼3 weeks until the pools were fully recovered with >90% viability. Protein production was carried out by inoculating a nutrient-rich medium with 2 × 10^6^ cells/ml and incubating at 36 °C in 5% CO_2_ for 7 days. Culture media were harvested from the production by centrifugation (1,000 × *g*, 5 min) followed by 0.22-μm sterile filtration. Protein concentration expressed in the harvested media was determined with an Octet RED96 (ForteBio, Menlo Park, CA) using Protein A biosensors according to the manufacturer's recommendations.

##### Large Scale Production of Agonistic Monoclonal Antibodies (mAbs)

For large scale production of antibody 27C3, antibody-expressing CHO-K1 cells were scaled to 20 liters in a Wave bioreactor (GE HealthCare) along with a 1-liter satellite shake flask. Standard fed batch production conditions were used, including a seeding density of 2 × 10^6^ cells/ml at 36 °C and 5% CO_2_, and cells were harvested on day 7. Anti-human LCAT mAb 27C3-huIgG2 was captured from harvested cell culture fluid using MabSelect SuRE affinity resin (GE Healthcare). Antibody 27C3 was bound to resin and washed with buffers. Antibodies were subsequently eluted with 100 mm acetate (pH 3.5). Elution pools were adjusted to pH 5.0. MabSelect SuRe eluent was diluted 1:3 with Milli-Q water prior to loading onto Fractogel® EMD SO3 (EMD Millipore) resin equilibrated in 100 mm acetate (pH 5.0). 27C3 was bound to resin and washed with 100 mm acetate (pH 5.0). Protein was subsequently eluted with a 20 CV gradient elution to 600 mm NaCl, 100 mm acetate (pH 5.0). Purified agonistic mAbs were concentrated and formulated into buffer containing 100 mm acetate and 9% sucrose (pH 5.0).

##### Affinity Determination

All experiments were performed using surface plasmon resonance analysis (Biacore assay, Biacore 3000 optical biosensor developed by Biacore AB, Uppsala, Sweden). The following reagents were purchased from GE HealthCare: the HBS-EP running buffer, CM5 sensor chip, coupling reagents (*N*-ethyl-*N*′-(3-dimethylaminopropyl)-carbodiimide hydrochloride/*N*-hydroxysuccinimide), ethanolamine. Fc fragment-specific goat anti-human IgG was purchased from Jackson ImmunoResearch Laboratories (West Grove, PA). Biosensor analysis was conducted at 25 °C in HBS-EP running buffer (10 mm HEPES, pH 7.4, 150 mm NaCl, 3.0 mm EDTA, 0.05% surfactant P20). The CM5 sensor chip was conditioned with serial injections (30 s) of 0.1% SDS, 10 mm NaOH, 10 mm HCl and again with 0.1% SDS at 30 μl/min (first two injections) or 60 μl/min (last three injections). Fcγ fragment-specific goat anti-human IgG was prepared (30 μg/ml) in acetate 5.0 buffer (10 mm sodium acetate, pH 5.0) and immobilized (10,000 RU) to the sensor chip via standard amine coupling (*N*-ethyl-*N*′-(3-dimethylaminopropyl)-carbodiimide hydrochloride/*N*-hydroxysuccinimide) and ethanolamine blocking to flow cells 1–4. LCAT antibodies (150 ng/ml) were injected (10 μl) in sets of three over flow cell 2, 3, or 4 at a flow rate of 10 μl/min. This process captured 100 RU of antibody. After the capturing step, human (100 nm to 412 pm, with 3-fold dilutions) or cynomolgus (111 nm to 457 pm, with 3-fold dilutions) LCAT antigen was passed over flow cells 1–4 at a flow rate of 50 μl/min to observe the association (180 s) of antigen with each antibody. Each flow cell was then flushed with running buffer to observe the dissociation of human (300 s) or cynomolgus (180 s) antigen from the chip surface. Each concentration was tested in triplicate. Blank antigen injections (0 nm) were tested before, after, and intermittently throughout the duration of the experiment. The surface was regenerated (25 μl at 50 μl/min) with glycine 1.5 (10 mm glycine-HCL, pH 1.5). The data were analyzed with Scrubber2 (BioLogic Software, Campbell, Australia) as follows. Data from flow cell 1 (blank reference) was subtracted from the data from flow cells 2–4 (assay flow cell). These reference-subtracted data (2-1, 3-1 or 4-1) were then subtracted (double-referenced) from the corresponding 0 nm concentration data. The dissociation rate constant (*k_d_*) was fit to a 1:1 binding model and used as a fixed parameter to determine the association rate constant (*k_a_*). The equilibrium dissociation constant (*K_D_*) was determined from the equation, *K_D_* = *k_d_*/*k_a_*.

##### Crystallography of LCAT and Antibody Complex

Full-length human LCAT protein (aa 1–416) harboring mutations of L4F and N5D (36) and a tobacco etch virus-cleavable C-terminal His tag was expressed using the BacMam expression system (Thermo Fisher Scientific) in HEK293S cells. Protein was purified by affinity, ion exchange, and size exclusion chromatography. Tobacco etch virus protease was used to remove the His tag during the purification process. The 27C3 antibody with a caspase-3 cleavage site engineered on the heavy chain between the Fab and Fc domains was expressed in HEK293–6E cells. The 27C3 Fab was isolated by purification following cleavage with caspase-3. We found that the LCAT-27C3 complex did not crystallize, so we added a previously discovered tool Fab (Fab1) to assist in crystallization (37). Fab1 was expressed and purified from *E. coli*. Ternary complex was made and purified by size exclusion chromatography. The purified ternary complex was washed in 10 mm Tris (pH 7.5), 25 mm NaCl and concentrated to 8 mg/ml for crystallization. Diffraction quality crystals were grown with the LCAT-27C3-Fab1 complex from 0.1 m Hepes (pH 7.0), 5% PEG 20000. Crystals were taken to the Berkeley Advanced Light Source beamline 5.0.2, and a 2.45 Å data set was collected. Images were processed with iMOSFLM (38) and Aimless (39) from the CCP4 Program Suite (40). The LCAT-27C3-Fab1 crystals grow in the P2_1_2_1_2_1_ space group with *a* = 57.9, *b* = 127.6, and *c* = 256.1 Å with ∼60% solvent and one complex in the asymmetric unit. The crystal structures of the LCAT-Fab1 complex (37) and 27C3 Fab (2.05 Å structure, data not shown) were used as starting models for molecular replacement in Phaser (41). The initial model was built with multiple rounds of model building in Quanta (Accelrys) and refinement with CNX (42). Final model building and refinement were performed using Coot (43) and PHENIX (44), respectively. Validation with MolProbity (45) shows an overall score of 2.15 with 93.7% of the amino acids in Ramachandran favored regions. The final model has an *R* of 18.9% and an *R*_free_ of 24.2%. Structure figures were made using PyMOL (PyMOL Molecular Graphics System, Schrodinger, LLC).

##### Preclinical Pharmacology Assessment

Naive male cynomolgus monkeys were maintained at an Association for Assessment and Accreditation of Laboratory Animal Care International (AAALAC)-accredited facility. All animal careprocedures and experimental procedures were approved by the institutional animal care and use committee. Cynomolgus monkeys were fed twice daily with 20% protein Primate Diet (Harlan Teklad, Indianapolis, IN). Water was provided *ad libitum* from Lixit waterers. Environmental controls for the animal room were set to maintain 70 ± 6 °F, 30–70% relative humidity, a minimum of 10–12 air changes/h, and 12-h light/12-h dark cycle.

After an overnight fast, sera were collected to determine baseline lipid levels. Monkeys were randomized based on baseline HDL-C levels and divided into two groups (*n* = 6/group). Monkeys were administered 30 mg/kg anti-KLH control antibody or the 27C3(S42A) agonistic antibody via a single intravenous bolus. Serum samples were collected after overnight fasting, at various time points following dosing. LCAT-agonistic antibody concentrations in sera were determined using an immunoassay that detects total Fc with 50 ng/ml lower limits of quantification. The immunoassay was conducted as reported previously (46). Serum cholesterol levels were measured using a COBAS INTEGRA 400 plus analyzer (Roche Applied Science). ÄKTAexplorer with a Superose 6 10/300 GL filtration column (GE HealthCare) was used for fast protein liquid chromatography (FPLC) fractionation analysis of serum lipoproteins.

##### Examination of Endogenous LCAT Protein Mass in Serum Samples

Endogenous levels of LCAT protein were determined in both antibody-treated groups by pooling serum samples within a group for each selected time point. Prior to starting protein isolation, 3 μg/ml human Fc fragment-tagged rLCAT (rLCAT-Fc) protein was added to the pooled serum samples for use as an internal standard. Both endogenous serum LCAT and internal standard (LCAT-Fc) in 250-μl serum samples were isolated by binding for 1 h to 30 μl of phenyl-Sepharose, which had been equilibrated with binding buffer containing 30 mm HEPES (pH 7.6) and 150 mm NaCl. After binding, phenyl-Sepharose resins were recovered by centrifugation and extensively washed with the binding buffer. LCAT proteins captured on the resin were eluted with 100 μl of 2× SDS-PAGE sample buffer (reducing). 20 μl of the collected eluent was analyzed on a 4–20% SDS-PAGE. Recovered LCAT was detected by Western blotting with an anti-human LCAT antibody (9B14).

##### Statistical Analysis

Results of all measurements are expressed as the mean ± S.E. All statistical tests were evaluated at a significance level of α = 0.05. A two-way analysis of variance model was applied, and the *p* value was adjusted using Bonferroni correction. Comparisons were deemed statistically significant if the *p* value was <0.05.

## Results

### 

#### 

##### Generation and Identification of Fully Human Anti-human LCAT-agonistic Antibodies

**FIGURE 1.**
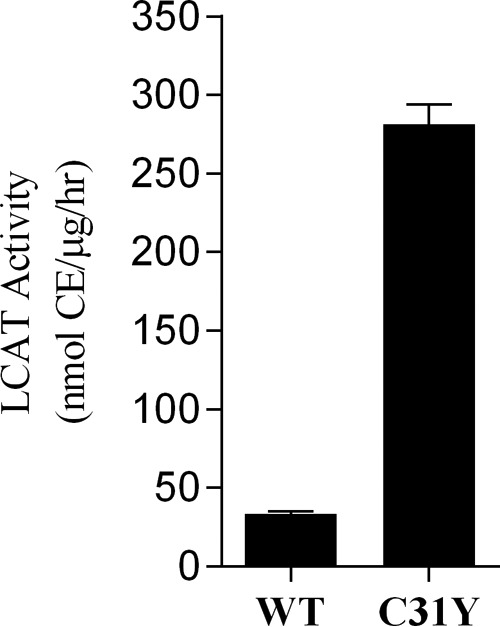
**Enzyme activities of purified recombinant human LCAT proteins.** Cholesterol esterification activities of recombinant wild type human LCAT (*WT*) and modified human LCAT (*C31Y*) proteins were determined at a protein concentration of 0.1 μg/ml in the reaction mixture as described under “Experimental Procedures.” This LCAT activity assay involves the use of human apoA-I/cholesterol/phospholipids liposomes as substrates and measures the conversion of radiolabeled FC to CE at 37 °C. FC and CE were separated and quantified by TLC. The experiment was performed multiple times, and the values shown are means ± S.E. (*error bars*) of measurements made from 10 representative experiments.

We used XenoMouse^TM^ technology to generate fully human mAbs against human LCAT by immunizing XenoMouse^TM^ with either the wild type rLCAT or a modified form of human LCAT protein with a single C31Y mutation (rLCAT(C31Y)). The rLCAT(C31Y) protein, which was characterized previously (28, 36, 37, 47), has ∼10-fold higher cholesterol esterification activity compared with wild type LCAT *in vitro* (Fig. 1).

**FIGURE 2.**
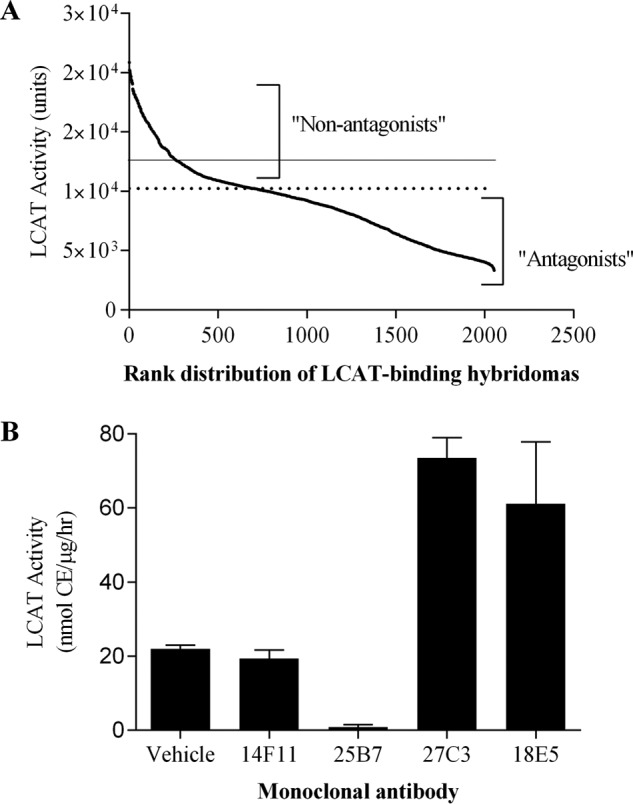
**Activities of LCAT-binding hybridomas and representative mAbs.** *A*, activities of LCAT-binding hybridomas on LCAT-mediated cholesterol esterification were determined by mixing individual hybridoma supernatant sample with purified rLCAT protein and the apoA-I proteoliposome substrates in the SPA format as described under “Experimental Procedures.” Hybridoma samples were tested in 384-well plates. 20 individual nonspecific control hybridoma samples that did not bind LCAT were tested under the same assay condition. The average activity of these non-binders is depicted as a *solid line* and serves as a baseline to differentiate agonistic antibodies from antagonists. All of the LCAT-binding hybridomas were ranked on the *horizontal axis* based on their LCAT activity units (cpm of [^3^H]CE), with the LCAT binders conferring highest activity being allocated to the *left* and those with lowest activity to the *right*. The hybridoma binders with LCAT activities lower than 1 S.D. of the average non-binder activity, as shown with a *dotted line*, were described as “antagonists” and eliminated from further analysis. The rest of hybridoma samples (ranked to the *left* with activities *above* the *dotted line*) were selected as “non-antagonists” and subjected to LCAT activity confirmation assays. *B*, activity of selected anti-LCAT mAbs on LCAT-mediated cholesterol esterification was determined by incubating purified individual mAb at 10 μg/ml concentration with purified native human LCAT proteins at 0.1 μg/ml concentration in a reaction mixture containing human apoAI-liposomes as the substrate. The assay was conducted as described in the legend to Fig. 1 and under “Experimental Procedures.” Values shown are means ± S.E. (*error bars*).

From the XenoMouse^TM^ hybridoma campaign, 1,748 hybridomas demonstrated binding to wild type human LCAT protein by ELISA. These hybridoma supernatants were screened for functional activity using an assay that measures LCAT-mediated cholesterol esterification. Because the majority of these hybridoma binders were presumed to be antagonistic, the primary screening assay was designed to eliminate the antagonistic antibodies. We developed a high throughput SPA to measure LCAT activity of the hybridoma samples in a reaction mixture containing LCAT enzyme and apoA-I/cholesterol substrates. Of the 1,748 LCAT-binding hybridomas, 258 hybridomas did not exhibit obvious antagonistic (inhibitory) effect on LCAT enzyme activity in comparison with a panel of nonspecific control hybridoma samples in the same assay (Fig. 2*A*). This group of 258 non-antagonistic hybridomas was subjected to rounds of subsequent analyses to identify agonistic antibodies that enhance LCAT enzyme activity. To this end, we performed LCAT activity assay using a more stringent TLC analytic format to confirm the effect of hybridomas on LCAT-mediated cholesterol esterification. We identified five hybridoma supernatants that considerably increased the enzymatic activity of the native human LCAT protein. Subcloning of these potential agonistic hybridomas led to monoclonal hybridomas. Two of the resultant mAbs from the five hybridomas were found to be identical upon sequencing, resulting in a total of four unique mAbs, including 27C3 and 18E5. All four agonistic mAbs were found to originate from the XenoMouse campaign that used the superactive mutant rLCAT(C31Y) protein as the immunogen. None of the mAbs from the immunogen of wild type LCAT protein exhibited agonistic activity. The four agonistic mAbs were purified from clonal hybridoma exhaust supernatants and retested to confirm activity. The effectiveness of purified 27C3 and 18E5 mAbs in agonizing human LCAT was compared with that of a neutralizing (inhibitory) antibody, 25B7, and another antibody, 14F11, that neither enhances nor inhibits LCAT activity. Both 25B7 and 14F11 were identified from the same XenoMouse campaign (Fig. 2*B*). All purified mAbs reproduced the activities of modulating LCAT that were originally observed in their parental hybridoma supernatants.

27C3 and 18E5 antibodies were chosen for further characterization based on their superior effectiveness in agonizing native human LCAT enzyme. Not only can the 27C3 antibody agonize the native human LCAT enzyme; it can also further enhance enzymatic activity of superactive human LCAT(C31Y) mutant enzyme. When tested for the activity of esterifying cholesterol using apoAI-proteoliposomes, 27C3 antibody increased LCAT(C31Y) activity ∼1.6-fold, whereas it increased the activity of native LCAT 3.0-fold under comparable experimental conditions. Binding affinity of each mAb to the native human LCAT and cynomolgus LCAT proteins was determined using surface plasmon resonance analysis (Biacore assay). Despite their distinct effects on modulating human LCAT enzymatic activity (Fig. 2*B*), the four human anti-human LCAT mAbs showed binding affinities that were comparable (Table 1). 27C3 showed a relatively weak binding affinity (*K_D_* = 80 nm) to cynoLCAT, whereas 18E5 showed no detectable binding. Neither 18E5 nor 27C3 binds to the LCAT of rodent or rabbit origin (data not shown).

**TABLE 1:** **Binding affinity of monoclonal antibodies to recombinant LCAT protein** Binding affinities of four mAbs to recombinant human and cynomolgus monkey (cyno)LCAT proteins were determined using surface plasmon resonance analysis as described under “Experimental Procedures.” *k_a_*, association rate constant; *k_d_*, dissociation rate constant; *K_D_*, *k_d_*/*k_a_*. ND, no detectable binding.

mAb	k*_a_*	*k_d_*	k*_D_*
	*m*^−*1*^ *s*^−*1*^	*s*^−*1*^	*nm*
**Binding with human rLCAT**			
25B7	2.12E + 05	4.75E − 04	2.24
14F11	ND	ND	ND
18E5	1.32E + 05	1.33E − 04	1.01
27C3	1.74E + 05	2.29E − 04	1.32

**Binding with recombinant cynoLCAT**			
25B7	3.99E + 05	4.07E − 04	1.02
14F11	7.42E + 04	5.24E − 04	70.6
18E5	ND	ND	ND
27C3	1.90E + 05	1.53E − 02	80.7

Amino acid sequence analysis showed that 27C3 antibody belongs to the human IgG2 subclass with λ light chain (Table 2). A possible *N*-linked glycosylation site was identified in the heavy chain CDR1 region of 27C3. In an effort to optimize 27C3 mAb for large scale production, a site-directed mutation at position 42 (S42A) of the amino acid sequence was introduced to abolish the *N*-linked glycosylation site. Experiments that assessed binding affinity to native human LCAT and activation by the 27C3 mutant, 27C3(S42A), showed that it was comparable with 27C3 (data not shown). Except for x-ray crystallographic studies, 27C3(S42A) was chosen to be used in subsequent studies.

**TABLE 2:** **The variable heavy (V_H_) and light (V_L_) chain sequences of two agonistic human anti-human LCAT monoclonal antibodies**

Monoclonal antibody	Chain	Amino acid sequence
27C3	V_H_	QVQLQESGPGLVKPSQTLSLTCTVSGASISSGGYNWSWIRQHPGKGLEWIGYIYYSGSTYYNPSLKSRVTISVDTSKNQFSLKLSSVTAADTAVYYCARERGYCSSTSCSRVMDVWGQGTTVTVSS
18E5	V_H_	QVQLVQSGAEVKKPGASVKVSCKASGYTFTGYYMHWVRQAPGQGLEWMGWINPNSGGTNYAQKFQGRVTMTRDTSISTAYMELNRLRSDDTAVYYCARGRWELYAFDIWGQGTMVTVSS
27C3	V_L_	SSELTQDPAVSVALGQTVRITCQGDSLRSYYASWYQQKPGQAPVLVIYGKNNRPSGIPDRFSGSSSGNTASLTITGAQAEDEADYYCNSRDNIGNHQVFGGGTKLTVLG
18E5	V_L_	EIVLTQSPGTLSLSPGERATLSCRASQSVSGSYLTWYQQKPGQAPRLLIYGASSRATGIPDRFSGSGSGTDFTLTISRLEPEDFAMYYCQQYGGSPPFTFGPGTKVDIKR

##### Characterization of LCAT-agonistic Antibodies

**FIGURE 3.**
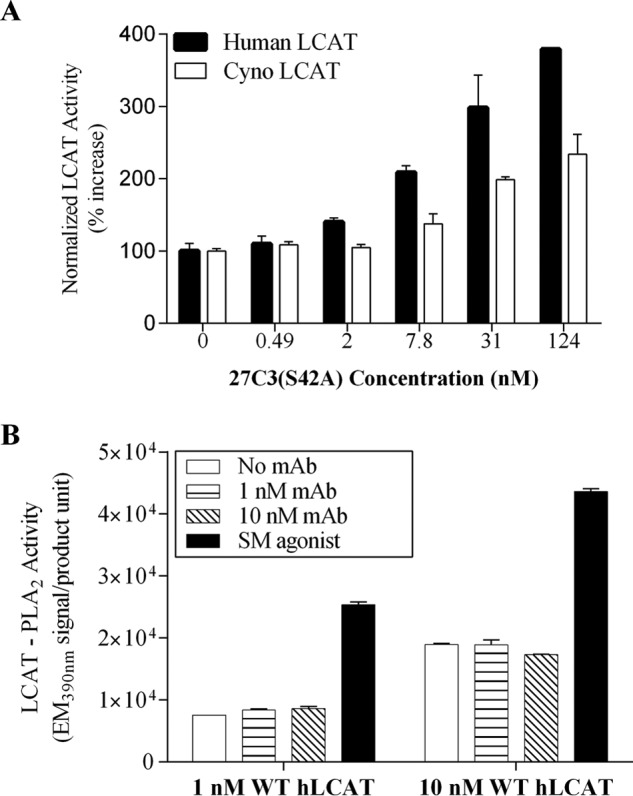
**Concentration-dependent activity of 27C3(S42A) on agonizing human LCAT and cynoLCAT enzymes *in vitro*.** *A*, activities of 27C3(S42A) antibody on enhancing the LCAT-mediated cholesterol esterification were determined by reacting various concentrations of antibody 27C(S42A) with either purified human native LCAT (*Human LCAT*) or with purified native cynomolgus monkey LCAT (*Cyno LCAT*). The assay conditions are described in the legend to Fig. 2*B* and under “Experimental Procedures.” Normalized LCAT activity refers to the percentage increase of the enzyme activity in comparison with its baseline enzyme activity in the absence of antibody. Values shown are means ± S.E. (*error bars*). *B*, the effect of antibody 27C3(S42A) on human LCAT-PLA_2_ activity was determined using a commercial assay kit as described under “Experimental Procedures.” Recombinant human wild type LCAT (*hLCAT*) protein was incubated with the 27C3(S42A) antibody (*Ab*) at the indicated concentrations prior to the addition of PLA_2_ substrates. The LCAT-PLA_2_ activity was determined by measuring hydrolyzed product-derived emission at 390 after a 4-h incubation at 37 °C. A small molecule agonist of LCAT (*SM agonist*) was tested at 3 μm. Values shown are means ± S.E.

Binding affinity measurement showed that 27C3(S42A) mAb binds to human and cynomolgus monkey native LCAT proteins; however, binding to native human LCAT is 61-fold higher (Table 1). In an LCAT activity assay that measures cholesterol esterification using apoA-I/liposomes as substrates, 27C3(S42A) enhanced the activities of both human and cynomolgus monkey native enzymes in a dose-dependent manner. Fig. 3*A* shows a bar graph comparison of the activities of 27C3(S42A) in agonizing human *versus* cynomolgus monkey native LCAT enzymes. It is apparent that 27C3(S42A) is more active on the native human LCAT than on the cynomolgus monkey enzyme. Although we were not able to precisely quantitate this apparent difference, this finding suggests that there might be a correlation between binding affinity and the LCAT-agonizing activity.

In an attempt to elucidate the mechanism by which agonistic mAbs enhance LCAT-mediated cholesterol esterification, we examined LCAT-phospholipase A_2_ (PLA_2_) activity, a reaction thought to be the first step and also the putative rate-limiting step of LCAT-mediated cholesterol esterification that precedes the LCAT-acyltransfer step (48). We found that 27C3(S42A) had no effect on LCAT-PLA_2_ activity even at 10-fold molar excess to LCAT protein in the reaction (Fig. 3*B*). Adding to the reaction with 12 μg/ml apoA-I did not alter LCAT-PLA_2_ activity (data not shown). In identical assay conditions, a previously identified small molecule LCAT agonist (29, 35) increased LCAT-PLA_2_ activity up to 3-fold, a magnitude consistent with that of small molecule agonist in its activity of increasing the LCAT-mediated cholesterol esterification rate.

**FIGURE 4.**
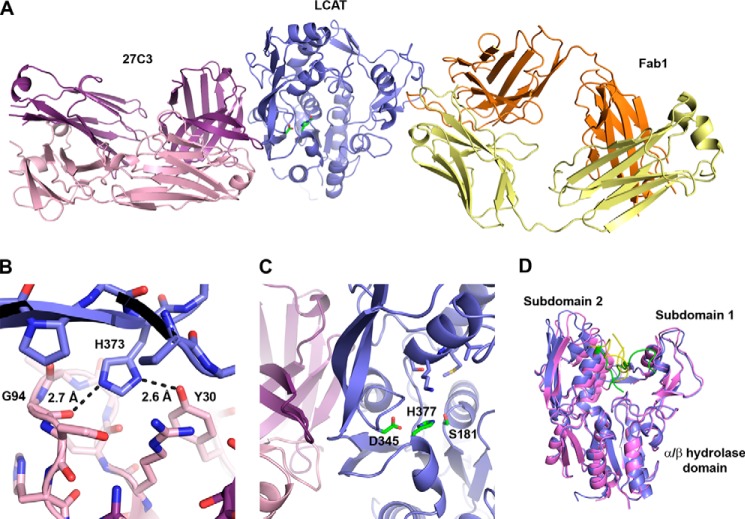
**Crystal structure of 27C3 bound to the human LCAT protein.** *A*, overall structure of the complex in a graphic depiction. LCAT is shown in *blue* in the *center* with the catalytic triad *highlighted* in *green sticks*. The agonist 27C3 is bound on the *left-hand side* of LCAT with the light chain *colored pink* and the heavy chain *colored purple*. The tool Fab1 is bound on the *right-hand side* with the light chain *colored yellow* and the heavy chain *colored* orange. *B*, figure highlighting the only difference between human and cynomolgus monkey LCAT at the 27C3 interaction interface (H373R). H373 from human LCAT makes two hydrogen bonds with the light chain of 27C3 and is buried under the interface between the two molecules. The larger arginine amino acid from cynomolgus monkey LCAT cannot form these two hydrogen bonds. *C*, *close-up view* at the interaction site between LCAT and 27C3. LCAT catalytic triad amino acid residues are shown as *green sticks* and *labeled*, and the amino acids from subdomain 2 that make up the back pocket of the substrate binding site are shown as *blue sticks*. 27C3 binds directly behind catalytic amino acid Asp-345 and subdomain 2 and may increase LCAT activity through stabilization of the catalytic triad and substrate binding site in an active conformation. *D*, superposition of the LCAT proteins from the 27C3-bound (*slate*) and free (*violet*; Protein Data Bank entry 4XX1) complexes with lid regions *highlighted* (*yellow* and *green*, respectively). The conformational shift of the lid region and the slight opening between subdomains 1 and 2 can be seen.

To further understand the molecular basis of the protein-protein interaction between 27C3 and native human LCAT, we solved the crystal structure of 27C3-bound LCAT protein to 2.45 Å resolution (Fig. 4*A* and Table 3). The interaction between 27C3 and native human LCAT buries ∼1,760 Å^2^ of total surface area and is composed of multiple hydrogen bonds and van der Waals interactions. Of the LCAT amino acids that make up the binding interface, there is a single amino acid difference between the human and cynomolgus monkey LCAT proteins (H373R). His-373 forms two hydrogen bonds with the light chain of 27C3 and is buried within the LCAT-27C3 interface (Fig. 4*B*). The corresponding arginine in native cynomolgus monkey LCAT protein (Arg-373) could not form identical interactions, thus potentially explaining a weaker binding affinity and a weaker agonizing activity of this monoclonal antibody with the native cynomolgus monkey LCAT (Table 1 and Fig. 3*A*). In addition, the crystal structure shows that 27C3 interacts with amino acids from both the α/β hydrolase core and subdomain 2 of native human LCAT protein in a region directly behind catalytic amino acid Asp-345 (Fig. 4*C*). The overall structure of native human LCAT complexed with 27C3 closely resembles the structure of native human LCAT in the LCAT-Fab1 complex described previously (Protein Data Bank entry 4XX1, LCAT Cα root mean square deviation = 0.590) (37). The 27C3 binding site and the surrounding regions on native human LCAT are remarkably similar with and without 27C3 bound. The most notable variations between the free native human LCAT and LCAT-27C3 complex can be seen in the flexible lid region (amino acids 225–248) and a slight increase in the separation between subdomains 1 and 2 (Fig. 4*D*), neither of which can be unambiguously attributed to the interaction with the 27C3 Fab. In this study, we also obtained a lower resolution structure of the LCAT(C31Y)-27C3 complex (data not shown). Similar to the observations with native enzyme structures, the LCAT(C31Y) mutant protein in the 27C3-bound form also appears to adopt the same structure as 27C3-free LCAT(C31Y) protein. Furthermore, we recently reported that the native LCAT and the superactive LCAT(C31Y) mutant enzymes superpose quite well to each other, indicating that the C31Y mutation does not cause a significant change to the structure of the LCAT protein (37), whereas the mutation makes the enzyme about 10-fold more active (Fig. 1).

**TABLE 3:** **Data collection and refinement statistics for LCAT-27C3-Fab1** ASU, asymmetric unit; RMSD, root mean square deviation.

Parameter	Value
**Data collection**	
Wavelength (Å)	1.0000
Space group	P212121
Cell dimensions	
*a*, *b*, *c* (Å)	57.94, 127.59, 256.08
α, β, γ (degrees)	90, 90, 90
Resolution (Å)	40–2.45 (2.51–2.45)*^a^*
Completeness	93.5 (85.1)
Redundancy	5.7 (5.7)
*R*_merge_	0.114 (1.268)
*CC*½	0.997 (0.612)
*I*/σ*I*	12.2 (1.6)

**Refinement**	
Resolution (Å)	30–2.45
Complexes/ASU	1
Reflections	
Total	66,114
Working set	62,890
Test set	3,224
*R*_work_/*R*_free_	0.189/0.242
RMSDs	
Bond lengths (Å)	0.010
Bond angles (degrees)	1.306
Protein Data Bank code	5BV7

*^a^* Numbers in parenthesis are for the highest resolution shell.

##### Pharmacology of the LCAT-agonistic Antibody in Non-human Primates

**FIGURE 5.**
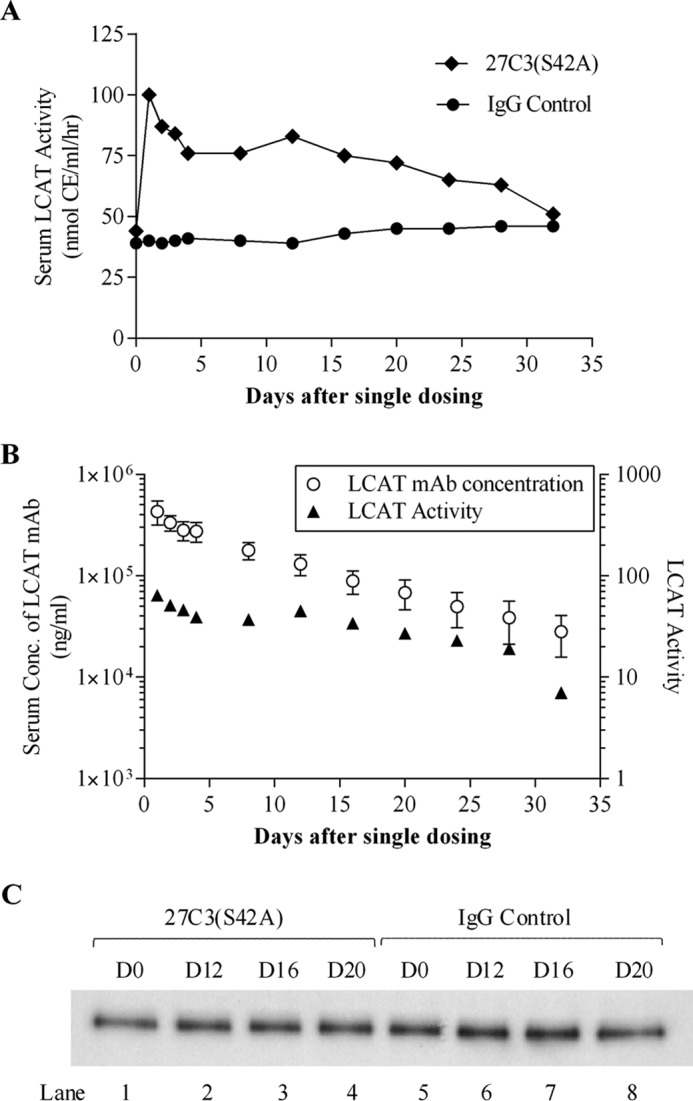
**27C3(S42A) activity on serum endogenous LCAT in cynomolgus macaques.** Male cynomolgus monkeys were randomized based on their baseline HDL-C levels and divided into two groups (*n* = 6/group). The animals were administered a single intravenous dose of either 30 mg/kg anti-KLH control (*IgG Control*) or 27C3(S42A) antibody as described under “Experimental Procedures.” Blood samples were collected after an overnight fast. *A*, serum LCAT activity was determined by incubating an aliquot of pooled serum samples from each group at each indicated time point with reconstituted human apoA-I liposome substrates and using TLC to quantify conversion of FC to CE (see “Experimental Procedures”). Each data point represents the mean of duplicate tests. *B*, serum concentration of 27C3(S42A) antibody (ng/ml) after single dosing and correlation between serum concentration of 27C3(S42A) and serum LCAT enzyme activity (nmol of CE/ml/h). Values of serum concentration of 27C3 mAb shown are means ± S.E. (*error bars*). *C*, the endogenous LCAT protein levels in cynomolgus monkey sera collected at different days after dosing (day 0 (*D0*) up to day 20 (*D20*)) were examined by Western blotting as described under “Experimental Procedures.”

**FIGURE 6.**
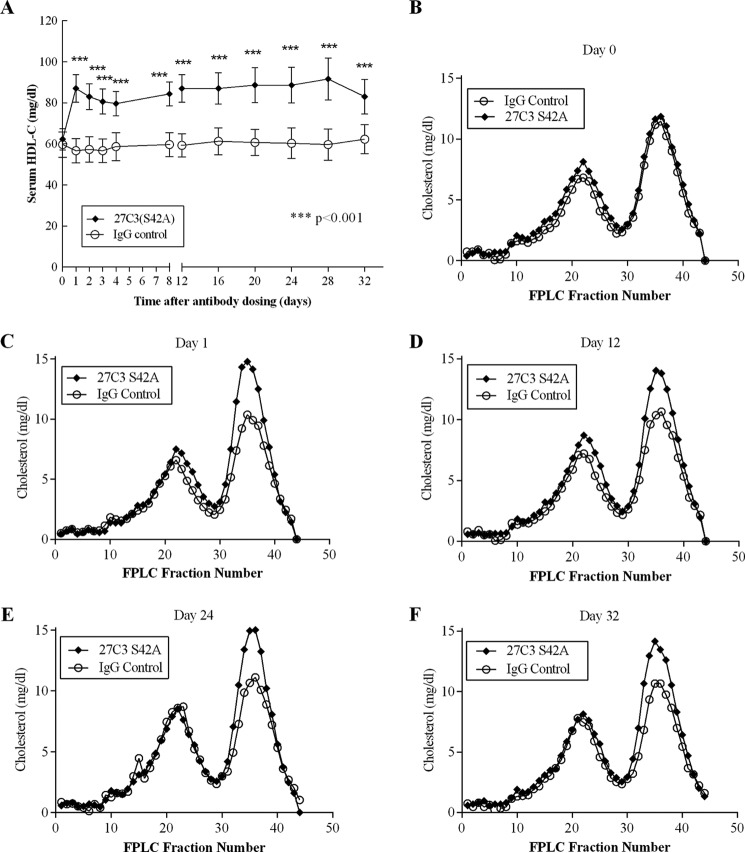
**Modulation of HDL metabolism by 27C3(S42A) in cynomolgus monkeys.** Randomized cynomolgus monkeys were divided into two groups (*n* = 6/group) and administered a single intravenous dose of either 27C3(S42A) or anti-KLH control (*IgG Control*) antibody as described in the legend to Fig. 5. *A*, serum concentrations of HDL-C were measured using a Cobas Integra 400 chemistry analyzer. Values shown are means ± S.E. (*error bars*). The increases of plasma levels of HDL-C in the 27C3(S42) treatment group are statistically significant across all of the test points in comparison with the control animal group at the same test time point (*p* < 0.001). *B–F*, serum lipoprotein profile at five selected time points over the course of the study was determined by gel filtration using FPLC to fractionate serum lipoprotein particles as described under “Experimental Procedures.”

*In vivo* studies were conducted in cynomolgus monkeys to evaluate the effects of 27C3(S42A) on the blood circulating LCAT as well as pharmacokinetic and other pharmacological properties. Due to its relatively weak binding affinity to cynomolgus monkey native LCAT and a high concentration of endogenous LCAT protein in the animals, 30 mg/kg 27C3(S42A) agonistic antibody was administered as a single intravenous dose to a group of six monkeys after randomization of the animals in the study. A nonspecific anti-KLH control IgG antibody was administered similarly to six monkeys as a control. Blood samples at multiple time points over 32 days after dosing were collected for analyses (Figs. 5 and 6). 24 h after dosing 27C3(S42A), a 2.3-fold increase of the serum LCAT enzyme activity was observed, followed by a steady elevation of LCAT activity that was sustained for ∼20 days before gradually returning to baseline levels (Fig. 5*A*). Over the course of the study, there was a correlation between increased LCAT enzymatic activity and 27C3(S42A) serum concentration (Fig. 5*B*). To evaluate whether 27C3(S42A) treatment altered levels of circulating LCAT protein, we measured LCAT protein mass in the serum samples collected over the course of antibody treatment. Comparable serum levels of endogenous LCAT proteins were present in the 27C3(S42A)-treated monkeys and the anti-KLH control IgG-treated monkeys at every time point measured (Fig. 5*C*). This result suggests that the increase of serum LCAT activity only observed in the 27C3(S42A)-treated group was not the result of an increase in serum LCAT protein mass.

To determine whether LCAT agonism by 27C3(S42A) translated to an increase of plasma HDL-C levels, we measured serum lipids and lipoproteins in 27C3(S42A)-treated and anti-KLH control IgG-treated monkeys. A single dose of 27C3(S42A) resulted in an ∼35% increase in serum HDL-C level at the day 1 time point over control animals (Fig. 6*A*). The increase of HDL-C levels was maintained for over 32 days and was statistically significant different (*p* < 0.001) from control for all of the time points measured (Fig. 6*A*). There were no significant changes in either the non-HDL cholesterol levels or apoB measurements over the study period (data not shown). FPLC fractionation confirmed the increases in HDL species in the 27C3(S42A)-treated group at every time point collected over the duration of the study (Fig. 6, *B–F*). The FPLC profile also confirmed no significant changes in the LDL or VLDL species.

## Discussion

Whereas antagonists represent a large class of biopharmaceutical therapies, many validated and tractable drug targets require an agonist-based approach to achieve desired pharmacological effects. Currently, small molecule agonists and recombinant proteins are the principal modalities for agonizing enzyme targets. However, fully human monoclonal antibodies are increasingly being recognized as an important therapeutic modality due to antibody-antigen specificity, potency, extended half-life, and safety profiles that are superior to those of other modalities (49). Because conventional antibody discovery efforts mostly generate blocking or neutralizing antibodies, agonistic antibodies capable of activating or enhancing the activities of target enzymes have rarely been identified. In this study, we harnessed an innovative immunization scheme and high throughput screening to search for agonistic monoclonal antibodies against a circulating enzyme, LCAT. In our search, we discovered four fully human agonistic mAbs.

Interestingly, all four agonistic mAbs were identified from a unique immunization and screening strategy based on two suppositions: 1) circulating LCAT enzyme can be activated by an agonistic antibody to achieve a greater enzymatic activity; 2) a modified form of LCAT protein with enhanced enzymatic activity might increase the likelihood of generating agonistic antibodies that bind and agonize the native enzyme. These suppositions stemmed from our previous studies in which we identified a small molecule agonist that increased LCAT enzymatic activity 3–4-fold (29, 35). Thereafter, we engineered the human LCAT protein and developed a superactive human LCAT enzyme by introducing a point mutation of C31Y to the amino acid sequence. This single point mutation enhances LCAT enzymatic activity ∼10-fold greater than that of the native wild type enzyme in driving cholesterol esterification (Fig. 1) (28, 36). Thus, we used this modified superactive rLCAT(C31Y) protein as an immunogen to initiate a XenoMouse^TM^ antibody campaign in parallel with native wild type human LCAT protein. A novel high throughput screening assay, SPA, was developed to efficiently identify agonistic antibodies in hybridoma supernatants. This was accomplished by first identifying and excluding antibodies that demonstrated antagonistic properties, thus leaving only non-inhibiting and/or agonistic LCAT antibodies for follow-up analyses. We did not discover agonistic antibodies from a parallel immunization campaign using native wild type human LCAT protein as the immunogen. Although our sample size in this antibody campaign is relatively small for drawing definite conclusions, we speculate that using the modified superactive rLCAT(C31Y) protein as the immunogen may have contributed to the favorable outcome. This strategy of utilizing a modified superactive form of the target enzyme for immunization to generate agonistic antibodies may be a viable approach to a host of other enzyme targets where agonism is required to achieve desirable pharmacological effects.

Enzyme agonism, as opposed to well established antagonism, is poorly understood at the molecular and mechanistic level. Although the precise mechanism underlying 27C3-mediated LCAT agonism remains to be fully elucidated, our x-ray crystallographic studies shed light on the nature of LCAT and 27C3 interaction. The 27C3 binding site on native human LCAT is in close proximity to the LCAT catalytic triad (Fig. 4, *A–C*), suggesting that 27C3 binding may stabilize the catalytic region consisting of the catalytic triad and/or substrate binding site in an active conformation. Comparing the LCAT-27C3 crystal structure with the crystal structure of LCAT unbound to 27C3 (Fig. 4*D*) (37), we do not see evidence that the 27C3 antibody changes the LCAT protein into a notably “more active” conformation. However, in the LCAT-27C3 crystal complex, the LCAT protein does appear to adopt a more “open” structure with the hydrolase lid pulled away from covering the active site when compared with the 27C3-free LCAT protein reported recently (Fig. 4*D*) (37). This change, however, cannot be definitely attributed to the interaction with the 27C3 antibody, because the position of amino acid residues and secondary structure underneath the 27C3 antibody binding site are extremely similar between 27C3-bound and antibody-free LCAT proteins and may simply be due to the different crystal packing required to crystallize this complex. In our previous study, we examined and compared the structure of native human LCAT with that of the superactive LCAT(C31Y) mutant protein (37). Both the native LCAT and LCAT(C31Y) mutant proteins looked for all intents and purposes identical without evidence of conformational changes in the LCAT(C31Y) protein structure that could explain its 10-fold higher enzymatic activity. The similarity in protein structure between the native and C31Y mutant human LCAT enzymes may help to explain why the antibodies generated by the mutant protein are able to cross-react with native LCAT. In summary, we demonstrated that 1) the 27C3-bound and 27C3-free native LCAT proteins assume a similar structure; 2) the 27C3-bound and 27C3-free LCAT(C31Y) proteins assume a similar structure; 3) the native and the superactive C31Y mutant LCAT proteins also assume a similar structure (37); and 4) 27C3 is able to agonize both native wild type and the superactive C31Y mutant LCAT enzymes. These data collectively indicate that a notable change of LCAT protein structure may not be obligatory for the enzyme to achieve a greater activity. This structure analysis adds to the mystery and intrigue as to exactly why the C31Y mutant protein favored generation of agonistic antibodies.

LCAT-mediated cholesterol esterification is thought to be composed of three steps, including 1) initial binding of LCAT to the disc surface of HDL particles, 2) hydrolysis of the phosphatidylcholine *sn*-2 ester bond by the LCAT-PLA_2_ activity, and 3) transfer of the acyl group to cholesterol to complete cholesterol esterification. It was suggested that the hydrolysis of the phosphatidylcholine represents a rate-limiting step of the overall LCAT-mediated cholesterol esterification process (50). We thus performed a biochemical assay to determine whether the agonistic antibody affects LCAT-PLA_2_ activity. This activity was determined using a commercial kit that does not have apoA-I in the reaction. Data in Fig. 3*B* show that 27C3(S42A) did not change LCAT-PLA_2_ activity. In contrast, in the same assay experiment, a small molecule that activates LCAT in a mechanism mimicking the C31Y mutation increased LCAT-PLA_2_ activity up to 3-fold (Fig. 3*B*), which is largely in line with the activity of the small molecule agonist on increasing LCAT-mediated cholesterol esterification rate. This result suggests that the mechanism of LCAT agonism by 27C3 does not involve acceleration of LCAT-mediated hydrolysis of phosphatidylcholine; thus, LCAT activation by 27C3 is mechanistically distinct from that of the C31Y mutation and the small molecule agonist. This result from the LCAT-PLA_2_ assay does not inform whether apoA-I is critical for 27C3 to agonize LCAT, because apoA-I is not required for either the baseline LCAT-PLA_2_ reaction or the small molecule or C31Y-mediated activation on LCAT-PLA_2_. Adding apoA-I into the assay reaction did not change LCAT-PLA_2_ activity (data not shown). Investigating the subsequent step of acyltransferase activity of LCAT toward cholesterol esterification in the absence of apoA-I is challenging, because apoA-I is a structural determinant of the cholesterol-phospholipid substrate complex. Nonetheless, we attempted to formulate cholesterol and phospholipid substrates in apoA-I-free liposomes. LCAT enzymes tested in the absence of apoA-I failed to show a baseline activity on cholesterol esterification or an activity in any formulation with 27C3 antibody, small molecule agonist, or the C31Y activation strategies (data not shown). Together, these results indicate that the agonistic antibody does not serve as a surrogate for apoA-I in the activation of LCAT.

The data and information obtained from our studies collectively suggest a more unorthodox mechanism for LCAT agonism by the monoclonal antibodies. This mode of LCAT agonism is apparently not through enhancing LCAT-PLA_2_ activity and not through altering LCAT structure to a notable degree. Possibly, the increase in LCAT activity by the agonistic antibody could be attributed to an affinity increase (or kinetic difference (*i.e.* slower off rate)) between LCAT and the HDL/apoA-I.

Limitations of this study include lack of the structure of LCAT enzymes in the presence and absence of 27C3 when LCAT is interacting with the HDL or apoA-I. Acquiring these data from x-ray crystallography has proven elusive, and such a mixed macromolecular association study is challenging by any other current methods.

We speculate that the agonistic antibody enhances LCAT activity through improving enzyme interaction with HDL/apoA-I and/or facilitating substrate access to the catalytic triad of the enzyme. This model predicts that the step of LCAT binding/penetration to HDLs is critical in controlling the overall efficiency of LCAT-mediated cholesterol esterification and thus presents a unique opportunity for pharmaceutical intervention to this target and the pathway. The precise influence of agonistic antibody binding on LCAT penetration to the HDL and/or interaction with apoA-I remains to be established. Further studies to fully elucidate the details of this initial step of LCAT reaction are warranted.

The discovery of an agonistic antibody capable of modulating HDL metabolism *in vivo* is particularly exciting in the context of developing human therapeutics. In non-human primates, a single dose of 27C3(S42A) led to a rapid and sustained elevation of the LCAT activity in sera (Fig. 5*A*) and a subsequent robust increase of HDL-C levels that lasted for at least 32 days (Fig. 6). This pharmacokinetic and pharmacologic profile of 27C3(S42A) mAb make it a compelling therapeutic lead for further development. Because 27C3 and other identified agonistic antibodies do not cross-react with rabbit or rodent LCAT species, we were unable to evaluate the impact of these antibodies on atherosclerosis in these well established preclinical disease models. However, our previous studies using recombinant modified LCAT(C31Y) proteins in rabbits have provided proof-of-concept evidence that LCAT activation facilitates cholesterol mobilization, promotes RCT *in vivo*, and attenuates atherogenesis under high fat diet-induced hypercholesterolemia (28). We believe that LCAT enzyme activity is the driving force behind this pharmacodynamic effect, whereas the modality and mechanism of enzyme agonism are only important in modulating the magnitude of LCAT activity. Therefore, we postulate that 27C3(S42A) represents a prototype LCAT antibody therapy for the treatment of dyslipidemia and cardiovascular disease, particularly for patients with clinical manifestations of low HDL syndrome and partial LCAT deficiency, including patients with renal disease. We propose that agonistic anti-human LCAT antibodies may be developed in combination with the newly developed anti- proprotein convertase subtilisin/kexin 9 antibody therapy. Such a revolutionary combination therapy might be able to concurrently reduce cholesterol loading to the vessel walls and promote cholesterol removal from preexisting atherosclerotic plaque lesions.

## Author Contributions

M. Z., J. D., M. S., and D. M. conceived and directed the study; R. N. G., S. M., J. T., R. Z., J. C., A. G., T. C., M. S., J. D., and M. Z. coordinated the antibody campaign and lead identification research; D. E. P. and N. W. designed and performed the crystallization study; P. F., S. M., S. S., M. B., M. L., J. T., A. G., and T. C. performed *in vitro* assays and analyzed the results; D. L. and J. C. coordinated and performed the animal study. M. Z., R. N. G., P. F., and D. E. P. wrote the paper. All authors reviewed the results and approved the final version of the manuscript.

## Footnotes

2 The abbreviations used are:

CVD: cardiovascular disease
CETP: cholesteryl ester transfer protein
HDL-C: HDL cholesterol
FC: free cholesterol
CE: cholesteryl ester
RCT: reverse cholesterol transport
LCAT: lecithin-cholesterol acyltransferase(s)
rLCAT: recombinant LCAT
cynoLCAT: cynomolgus LCAT protein(s)
SPA: scintillation proximity assay
TLC: thin layer chromatography
HC: heavy chain
LC: light chain
PLA_2_: phospholipase A_2_.
